# Assessing Precision in All-Ceramic Fixed Restorations: Unveiling the Marginal Fit Through Digital and Traditional Impressions—A Comprehensive Systematic Review and Meta-Analysis

**DOI:** 10.1055/s-0045-1804528

**Published:** 2025-05-02

**Authors:** Waleed M. S. Alqahtani, Salah A. Yousief, Amir Mohidin R. Demachkia, Mohidin R. Demachkia, Ali Barakat, Mohiddin R. Dimashkieh, Mahmoud Abdallah M. Mekkey, Ahmed Mohammed Sleem Abdelglel, Ahmed S. Waly, Rawana Mohammed Saieed Bamanie, Dalal Abdulaziz Alnafisah

**Affiliations:** 1 Department of Prosthetic Dentistry, College of Dentistry, King Khalid University, Abha, Saudi Arabia; 2 Department of Restorative and Prosthetic Dental Sciences, College of Dentistry, Dar Al Uloom University, Riyadh, Saudi Arabia; 3 Department of Crown and Bridge, Faculty of Oral and Dental Medicine, Al-Azhar University, Assiut Branch, Cairo, Egypt; 4 Department of Dental Materials and Prosthodontics, São Paulo State University (UNESP), São José dos Campos, Brazil; 5 Private Clinic, Lasting Smile, Riyadh, Saudi Arabia; 6 Fixed Prosthodontics Department, Faculty of Oral and Dental Medicine, South Valley University, Qena, Egypt; 7 Deprtment of Fixed Prosthodontics, Faculty of Dental Medicine, Al-Azhar University, Assuit Branch, Cairo, Egypt; 8 Division of Pediatric Dentistry and Dental Public Health, Department of Pediatric Dentistry, Faculty of Dentistry, Al-Azhar University, Assiut, Egypt; 9 General Dentist, KSA; 10 Dentist Resident, Ministry of Health, KSA

**Keywords:** marginal fit, restoration, ceramic, digital

## Abstract

The marginal fit of all-ceramic fixed restorations is critical to long-term success. This comprehensive study and meta-analysis assessed the marginal fit of restorations manufactured using digital versus conventional impression procedures. We conducted a comprehensive search of electronic databases such as PubMed, Cochrane CENTRAL, Web of Science, and Scopus for publications published up to 2023. Eligible papers comparing the marginal fit of all-ceramic permanent restorations made using digital and conventional impressions were considered. A total of 19 studies met the inclusion criteria. The pooled analysis revealed that restorations fabricated from digital impressions exhibited a significantly better marginal fit than those from conventional impressions and showed a mean marginal gap of –13.76 µm (95% confidence interval: [–24.77, –2.76], *p* -value = 0.01). Subgroup analysis by type of digital impression system demonstrated consistent superiority of zironica material over other ones. However, high heterogeneity was observed among the included studies ( *I* ^2^ = 90.74%). Digital impression restorations show superior marginal fit compared with conventional impressions, but high heterogeneity requires cautious interpretation and further well-designed studies to validate results.

## Introduction

In the 1800s, the initial dental impressions were made using wax, aiding in transferring tooth shapes to laboratories. This process proved invaluable to dentists and technicians, facilitating their work in creating accurate dental prosthetics and restorations. 1 2

Marginal fit refers to the degree of precision of the restoration to the prepared tooth structure at the margin interface. 3 4 An ideal marginal fit ensures that the restoration and the tooth are smoothly integrated, limiting microgaps and potential areas for plaque accumulation, and reducing the risk of additional caries and periodontal complications. The marginal fit of dental restorations, particularly ceramic fixed restorations, is critical because it determines the restoration's longevity and success. 3 5 6 7 8 9

Techniques like using computer-aided design/computer-aided manufacturing (CAD/CAM) systems with accurate impression methods play a significant role in achieving optimal marginal fit, maintaining a tight seal between the restoration and the tooth through proper fit and cement thickness. This helps to prevent dissolution of luting material and subsequent secondary caries. 3 6 10 11 12 13

In the past, dental restorations have been made using conventional impression techniques that involve the use of elastomeric materials to capture tooth preparations. It uses materials such as vinyl polysiloxane (VPS) or polyether, which are applied directly to the patient's teeth and soft tissues and then set to form a model. However, despite their efficacy, conventional impressions have drawbacks such as material shrinkage, deformation during removal from the oral cavity, and inaccuracies in tray seating that can compromise the fidelity of conventional impressions, leading to suboptimal marginal fit of the final restoration. 5 14 15 16

Digital impressions have emerged through the use of intraoral scanners to capture three-dimensional images of the oral cavity, which are subsequently transformed into computerized models that revolutionized the area of restoration overcoming the limitations of conventional methods providing more patient satisfaction and comfort. 15 17 18

Depending on the restoration's specific needs, various materials such as ceramics, metals, and polymers can be used in conjunction with both digital and traditional impressions. Each approach has advantages and disadvantages, and the decision between digital and traditional methods is frequently influenced by factors such as clinician preference, case complexity, and available technology. 15 19 20

The transition from conventional to digital impression techniques has sparked considerable interest among researchers and clinicians, prompting numerous studies to investigate the comparative efficacy of these approaches in terms of marginal fit and overall clinical outcomes. 8 20 21 22 23 All-ceramic restorations, valued for their esthetic properties, biocompatibility, and durability, have grown in popularity in modern dentistry, necessitating a thorough understanding of the factors influencing marginal fit. 15 24 25

This systematic review and meta-analysis examined the marginal fit of all-ceramic fixed restorations using digital and conventional impression techniques, providing insights for clinical decision-making and practice guidelines.

## Methods

### Study Selection

#### Literature Search Strategy

A comprehensive search strategy was employed to identify relevant studies from electronic databases including PubMed, Cochrane Library, Scopus, and Web of Science. The search was conducted up to 2023 with English language restrictions. We used Medical Subject Headings terms and keywords related to “fixed restorations,” “marginal fit,” “digital impressions,” and “conventional impressions” in combination to reach all related articles.

#### Eligibility Criteria

Studies were included if they met the following criteria:

- Comparative studies that evaluate the marginal fit of all-ceramic fixed restorations fabricated from digital and conventional impressions.
- Study designs including randomized controlled trials (RCTs), *in vitro* experiments, and *in vivo* studies were included.
- Studies report quantitative data on marginal fit measurements such as marginal gap.
- Studies published in peer-reviewed journals.

The exclusion criteria were as follows:

- Case reports, case series, reviews, and editorials.
- Studies that did not directly compare digital and conventional impression techniques.
- Studies lacking sufficient data for analysis.

#### Study Selection Process

Using Rayyan software, two independent reviewers assessed eligibility by screening titles and abstracts of identified records. We assessed full-text articles of potentially eligible studies for inclusion and resolved discrepancies through discussion or consultation with a third reviewer when needed.

### Data Extraction

Using Microsoft Excel, a uniform data extraction sheet was created to gather useful information from the included research. From the included studies, each author gathered the following information: the first author's name, the year of publication, the type of study, the number of enrolled teeth, the materials, the major findings, the specifics of the intervention, and the outcomes. We used the total number of restorations and not the patients in all included studies. Two senior authors reviewed the extracted data. Any disagreements were resolved by group discussion.

### Quality Assessment

For RCTs, the Cochrane Risk of Bias Tool 2 (ROB 2) was used to assess the methodological quality of the included research. For *in vitro* and *in vivo* studies, the Methodological Index for Non-Randomized research (MINORS) scale was specifically designed. MINORS scale ensures a comprehensive assessment of both *in vitro* and *in vivo* research, focusing on critical factors such as sample size determination, unbiased outcome assessment, and comprehensive methodological reporting. Two reviewers independently evaluated each study, and any disagreements were resolved through discussion. Robvis' Web site was used to generate the risk of bias summary and graph. 26 27 28

### Data Synthesis and Analysis

We used Stata version 17 to perform quantitative analysis. Random-effects models were utilized due to high levels of heterogeneity and to pool data across studies and calculate overall effect estimates with 95% confidence intervals (CIs). The primary outcome measure was the mean difference (MD) in marginal fit between restorations fabricated from digital versus conventional impressions. The *I* ^2^ statistic was used to measure the heterogeneity between the studies; values higher than 50% indicated significant heterogeneity. Subgroup analyses were performed to investigate potential sources of heterogeneity and examine the influence of different digital impression systems on marginal fit outcomes.

## Results

### Study Selection

**Fig. 1:**
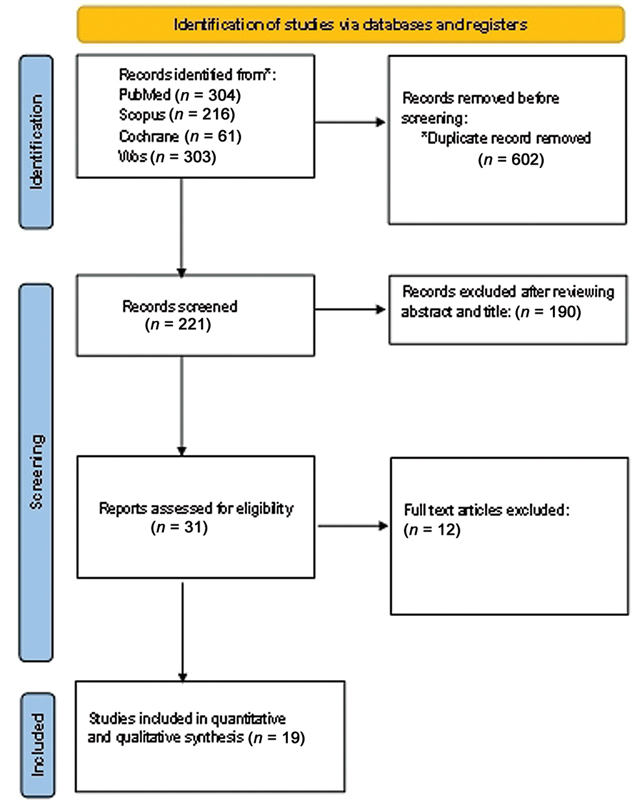
Preferred Reporting Items for Systematic Reviews and Meta-Analyses (PRISMA) flowchart.

The first database search produced 823 records in total. Thirty-one full-text papers were evaluated for eligibility following the removal of duplicates and the screening of titles and abstracts. In the end, 19 studies were included in the qualitative synthesis after meeting the inclusion criteria. A flowchart illustrating the study selection process is presented in Fig. 1 .

### Study Characteristics

There were eight RCTs, eight *in vitro* experiments, and three *in vivo* investigations among the included studies, representing a wide variety of study designs. Table 1 provides an overview of the features of the listed studies. The sample sizes in the studies ranged from 9 to 63 teeth in clinical studies and from 10 to 15 specimens in laboratory experiments. Different types of all-ceramic restorations were explored, including zirconia-based, lithium disilicate, and glass ceramic crowns, with digital impressions created using systems like Trios, Cerec Omnicam, and intraoral scanners, and conventional impressions using vinyl polyether silicone, VPS, and silicone rubber materials. The follow-up periods in clinical studies varied from short-term to long-term durations.

**Table 1:** Summary and baseline characteristics

Study ID	Experimental vs. control	Study design	Country	Number	Key finding	Material
Berrendero et al, 2016	Trios intraoral scanner vs. vinyl polysiloxane	RCT	Germany	30	Ceramic crowns fabricated using an intraoral scanner are comparable to elastomer conventional impressions in terms of their marginal and internal fits. The mean marginal fit in both groups was within the limits of clinical acceptability. Clinical significance Impressions based on ultrafast optical sectioning technology can be used for manufacturing ceramic crowns in a normal workflow, with the same results as silicone conventional impressions	Zirconia-based ceramic crowns
Boeddinghaus et al, 2015	Cerec Omnicam vs. vinyl polyether silicone	RCT	Germany	49 vs. 49	Within the limitations of this study, it can be concluded that zirconia copings based on intraoral scans and laboratory scans of a conventional model are comparable to one another about their marginal fit. Clinical relevance Regarding the results of this study, the digital intraoral impression can be considered as an alternative to a conventional impression with a consecutive digital workflow when the finish line is visible and it is possible to keep it dry	
	Trios vs. vinyl polyether silicone			49 vs. 49		
	3M Lava vinyl polyether silicone			50 vs. 49		
Gjelvold et al, 2016	Trios vs. vinyl polysiloxane	RCT	Sweden	24 vs. 24	The results of this study demonstrated that the digital technique was more efficient and convenient than the conventional impression technique	_
Rödiger et al, 2017	Cara Trios vs. vinyl polysiloxane	*In vivo*	Germany	20 vs. 20	CAD/CAM-fabricated zirconia single crowns produced with CI and IS techniques offer adequate marginal and internal precision. However, the IS technique provides lower internal gaps in some specific areas	Zirconia
Haddadi et al, 2019	Trios 3 vs. vinyl polysiloxane	RCT	Germany	10 vs. 9	Crowns based on IOS show statistically significantly better marginal and internal adaptation before cementation compared with conventional impressions. However, the clinical evaluation showed a similar marginal adaptation	Lithium disilicate crowns
Bosniac et al, 2019	Cerec AC Omnicam vs. vinyl polysiloxane	*In vivo*	Germany	63 vs. 63	The intraoral scanners tested allow for the production of single-tooth-restorations with an adequate marginal fit, whereas the production of restorations based on the scan of a conventional impression led to vast marginal gaps	Zirconia
	Cara Trios vs. vinyl polysiloxane					
Ahrberg et al, 2016	Lava C.O.S. vs. polyether	RCT	Germany	17 vs. 17	Although both direct and indirect digitalization facilitate the fabrication of single crowns and three-unit FDPs with the clinically acceptable marginal fit, a significantly better marginal fit was noted with direct digitalization. Digital impressions are also less time-consuming for the dental practitioner and the patient	Zirconia
Syrek et al, 2010	Lava C.O.S. vs. vinyl polysiloxane	RCT	Germany	9 vs. 9	Within the confines of the study the following can be concluded: 1. All-ceramic crowns resulting from intraoral scans with Lava C.O.S. demonstrated significantly better marginal fit than all-ceramic crowns fabricated from conventional two-step impressions 2. Marginal discrepancies in both groups were within the limits of clinical acceptability 3. All-ceramic crowns resulting from intraoral scans with Lava C.O.S. showed better interproximal contact point quality compared with all-ceramic crowns from conventional two-step impressions 4. Crowns from the two groups performed equally well with regard to occlusion	Zirconia
Pradíes et al, 2015	Lava C.O.S. vs. silicon	*In vivo* prospective clinical trial	Spain	33 vs. 33	Within the conditions and limitations of this study, it was concluded that zirconia-based ceramic crowns, when fabricated using digital impressions, exhibited superior marginal and internal fit compared with those fabricated using conventional impressions. Importantly, despite these differences, the mean marginal discrepancy observed in both groups fell within the bounds of clinical acceptability. This suggests that while digital impressions may offer advantages in terms of fit, both digital and conventional methods can achieve clinically acceptable results within the context of this study's parameters	Zirconia
Sakornwimon and Leevailoj, 2017	Digital vs polyvinyl siloxane	RCT	Thailand	8 vs. 12	No differences were found in the clinical marginal fit of zirconia crowns fabricated from either digital impression compared with PVS impressions. Furthermore, patients' satisfaction with digital impressions was significantly higher than with conventional impressions	Zirconia
Zarauz et al, 2016	Lava C + B16:B28OS vs. vinyl polysiloxane	RCT	Germany	26 vs. 26	All-ceramic crowns fabricated from intraoral digital impressions with parallel confocal technology demonstrated a clinically acceptable internal and marginal fit as a conventional impression. Clinical significance Intraoral digital impressions as initial step to the digital workflow could further improve the marginal adaptation of all ceramic single crowns	Zirconia
An et al, 2014	Tero with polyurethane vs. polyvinyl siloxane impression	*In vitro*	Korea	10, 10 vs. 10	The marginal gap between the restoration and definitive cast base metal die was greater in the groups that used the digital impression method than in the group that used the conventional impression method. However, the marginal discrepancies of all of the groups were clinically acceptable	Zirconia
	iTero with no dies vs. polyvinyl siloxane impression					
Seelbach et al, 2013	Cerec scan vs. single-step putty-wash impression and two-step putty-wash impression	*In vitro*	Germany	30 vs. 40	Can be stated that digital impression systems allow the fabrication of fixed prosthetic restorations with similar accuracy as conventional impression methods. Clinical relevance Digital impression techniques can be regarded as a clinical alternative to conventional impressions for fixed dental restorations	Empress CAD vs. Cera E alloy (20), Lava zirconia (20)
	Lava C.O.S. scan vs. single-step putty-wash impression and two-step putty-wash impression					Lava zirconia vs. Cera E alloy (20), Lava zirconia (20)
	iTero scan vs. single-step putty-wash impression and two-step putty-wash impression					Copran Zr-I vs. Cera E alloy (20), Lava zirconia (20)
Bandiaky et al, 2023	Trios 3 vs. polyvinyl siloxane	*In vitro*	France	27 vs. 27	The five-unit zirconia-based FDPs fabricated with digital scans showed better fit than those in the conventional impression group. Within the limitations of this study, these results are encouraging, and continued progress in the digital field should allow for more accurate long-span restorations	Zirconia
Liang et al, 2023	Intraoral scanner vs. silicone rubber		China	10 vs. 10	The digital measurement method for the absolute marginal discrepancy was preliminarily established based on open-source software and applied in three-unit ceramic fixed dental prostheses. The absolute marginal discrepancy of three-unit ceramic fixed dental prostheses fabricated using digital technology was comparable to that of conventional technique	Glass ceramic
Ng et al, 2014	Lava C.O.S. vs elastomer	*In vitro*	Canada	15 vs. 15	The fully digital fabrication method provided a better margin fit than the conventional method	Lithium disilicate glass
Anadioti et al, 2014	PVS/CAD/CAM (Group B) vs. PVS/Press (Group A)	*In vitro*	Iowa city	15 vs. 15	The combination of the PVS impression method and press fabrication technique produced the most accurate 3D and 2D marginal fits	Lithium disilicate
Kocaağaoğlu et al, 2019	Cdi cast scanning/Tdi digital impression vs. conventional	*In vitro*		20 vs. 10	The three-unit frameworks produced using digital impression methods exhibited superior marginal fit in comparison to those made with conventional impression techniques. All measured marginal misfit values were deemed clinically acceptable	CoCr W
Shembesh et al, 2017	iTero scan/Lava Definition/model scan vs. conventional	*In vitro*	Boston	30 vs.10	In this laboratory study, it can be inferred that the marginal gap observed with all impression methods remained within the clinically acceptable range of 120 µm. Among the groups tested, Group 4 (Lava True Definition) demonstrated the smallest average gap, followed by Group 2 (stone cast scan), Group 3 (Cadent iTero), and Group 1 (PVS impression scan), with statistically significant variances noted	Zirconia

Abbreviations: 2D, two-dimensional; 3D, three-dimensional; CAD/CAM, computer-aided design/computer-aided manufacturing; CoCr, cobalt-chromium; FDPs, fixed dental prostheses; IOS, intraoral scanner; PVS, polyvinyl siloxane; RCT, randomized controlled trial.

### Quality Assessment

**Fig. 2:**
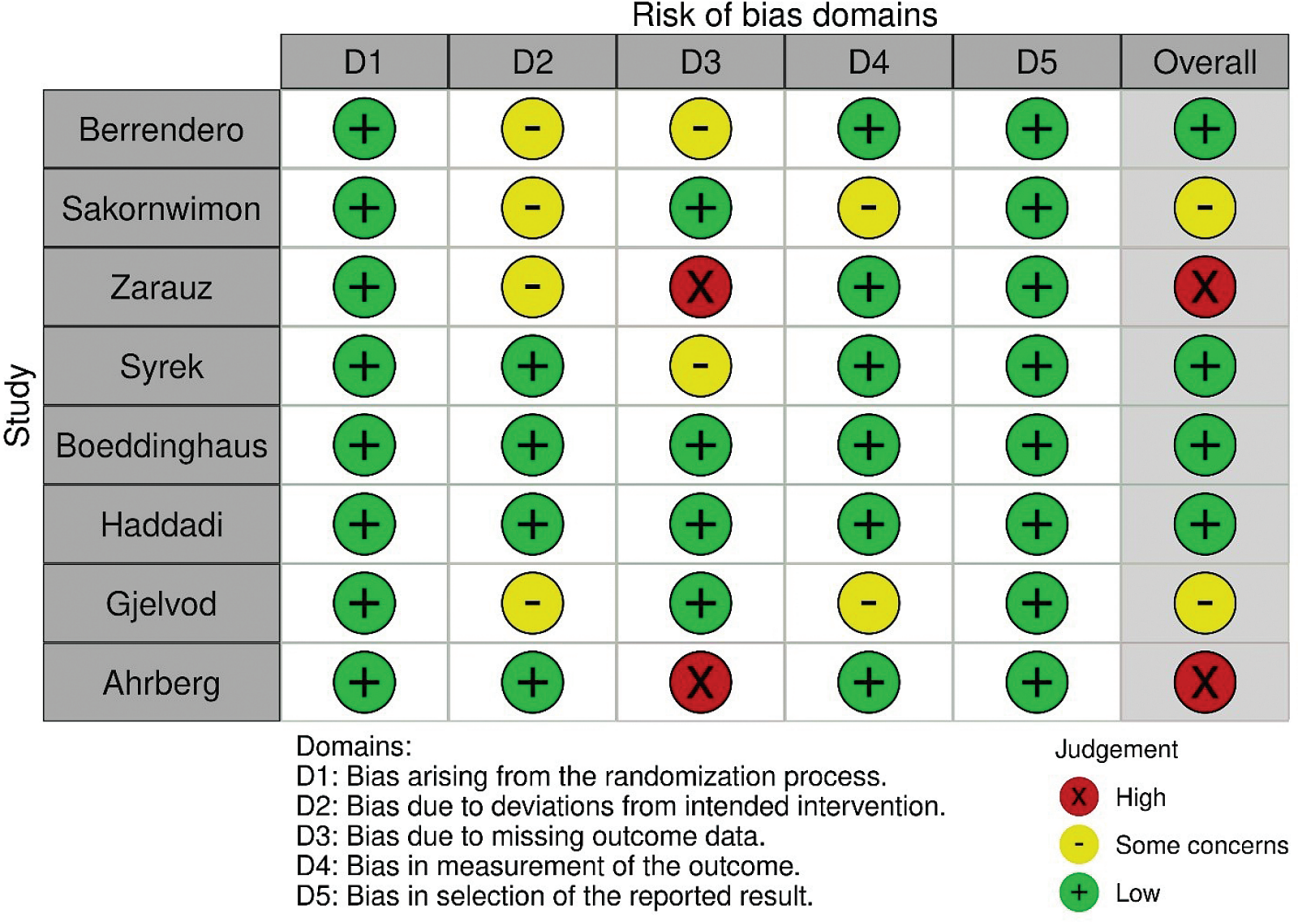
Cochrane Risk of Bias (ROB) summary.

**Fig. 3:**
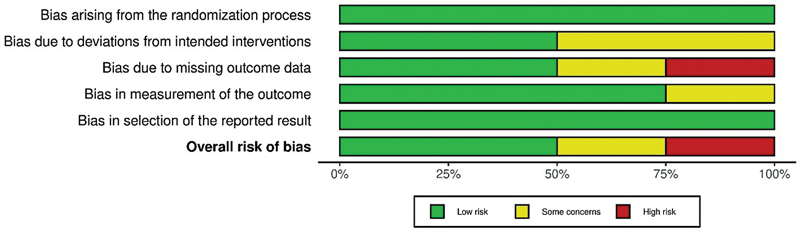
Cochrane Risk of Bias (ROB) graph.

Based on the MINORS scale and ROB-2 assessment, the overall methodological quality of the included studies was moderate to high. The majority of studies exhibited a low risk of bias or met essential quality criteria. However, two RCTs showed a high risk of bias, indicating the need for a cautious interpretation of its results ( Figs. 2 and 3 and Table 2 ).

**Table 2:** Risk of bias assessment

Evaluation	An et al (2014)	Anadioti et al (2014)	Ng et al (2014)	Seelbach et al (2013)	Bosniac et al (2019)	Rödiger et al (2017)	Pradíes et al (2015)	Bandiaky et al (2023)	Liang et al (2023)	Kocaağaoğlu et al (2019)	Shembesh et al (2017)
Clearly stated aim	2	2	2	2	2	2	2	2	2	2	2
Contemporary groups	2	2	2	2	2	0	2	2	0	2	1
Impression method	2	2	2	2	2	2	2	2	2	2	2
Control groups with other impression materials	2	2	2	2	2	0	2	1	0	1	2
Definitive restoration	1	2	2	2	2	2	2	2	2	2	1
Retentive element	1	1	1	1	1	2	2	2	2	2	2
Adequate number of observations	1	2	2	1	1	0	2	2	2	2	2
Preparation method	2	0	2	2	2	2	2	2	2	2	2
Power analysis	0	0	0	0	1	0	2	0	2	1	1
Statistical analysis	2	2	1	2	2	1	2	2	2	2	1
Prospective collection of data	–	–	–	–	–	–	2	2	2	2	2
Baseline equivalence of groups	–	–	–	–	–	–	2	1	1	1	2
Total score	15	15	16	16	17	11	24	20	19	19	18

### Outcome

**Fig. 4:**
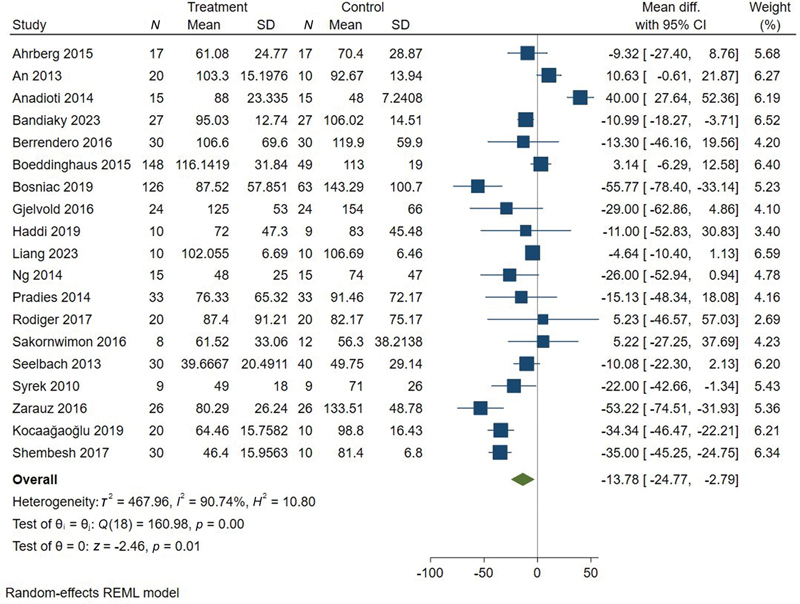
Meta-analysis of overall marginal fit.

A meta-analysis of 19 studies 7 8 9 11 12 13 20 21 22 23 29 30 31 32 33 34 35 36 37 was conducted to compare the marginal fit of all-ceramic fixed restorations fabricated from digital versus conventional impressions. The results of the meta-analysis are summarized in Figs. 4 5 6 7 8 9 .

**Fig. 5:**
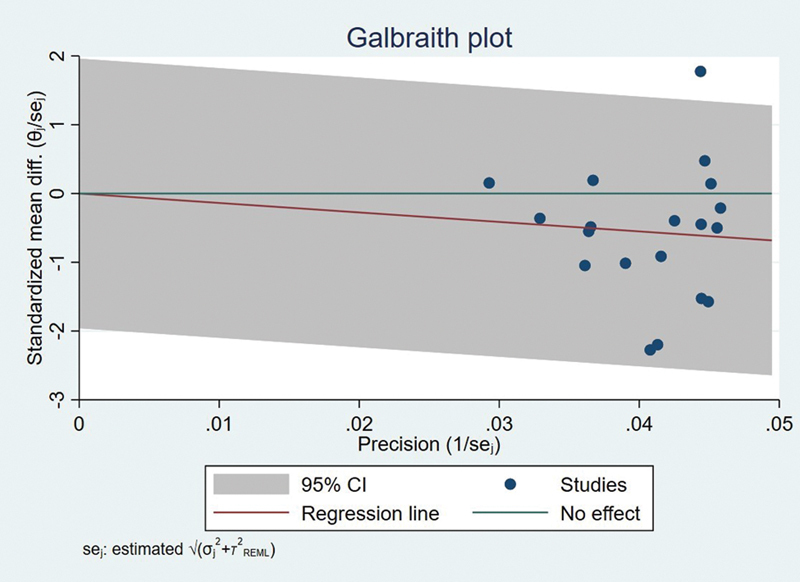
Galbraith plot.

**Fig. 6:**
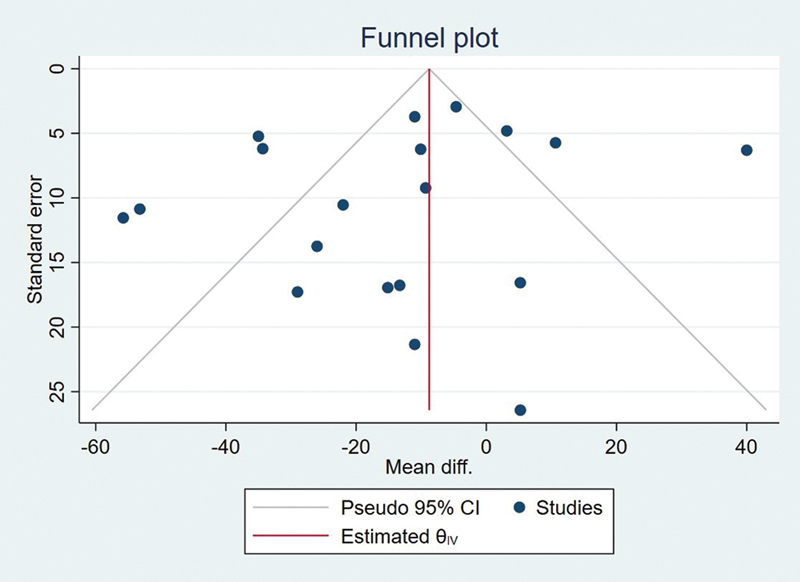
Funnel plot.

**Fig. 7:**
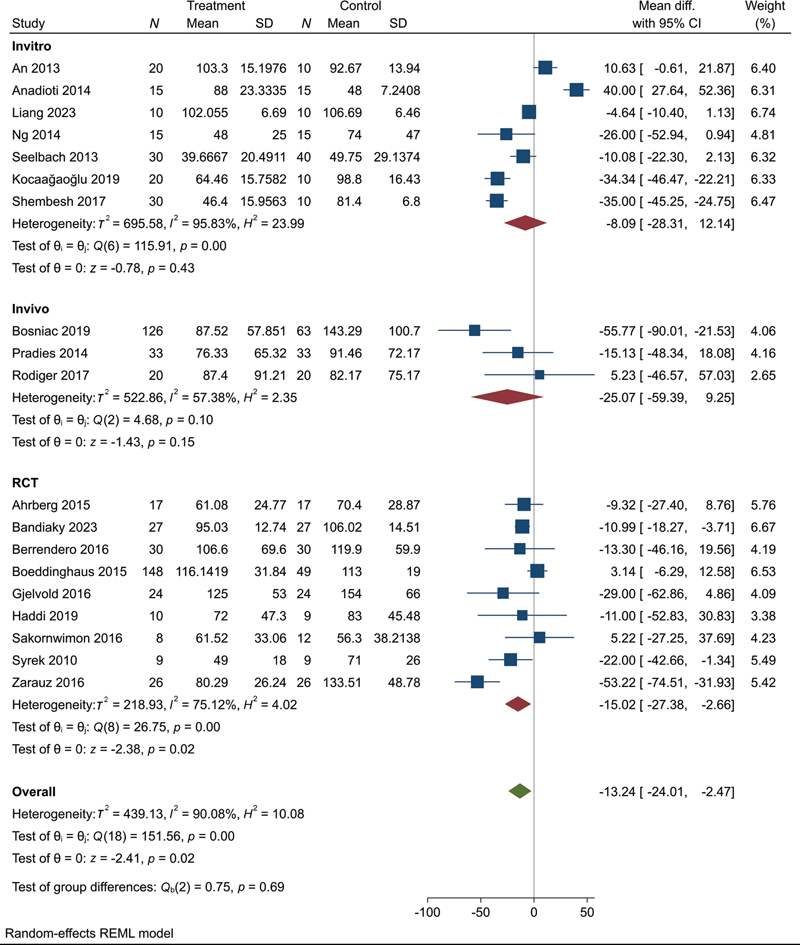
Subgrouping analysis according to study design.

**Fig. 8:**
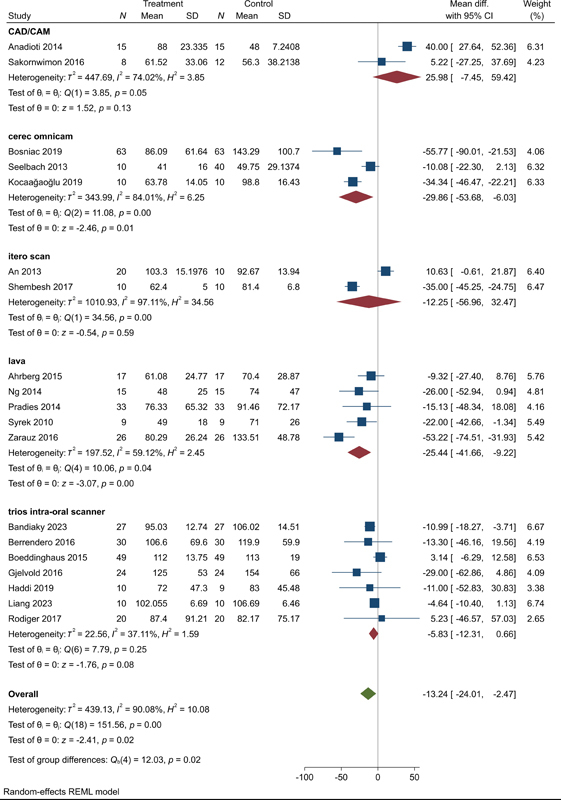
Subgroup analysis according to the technique of digital versus conventional impressions.

**Fig. 9:**
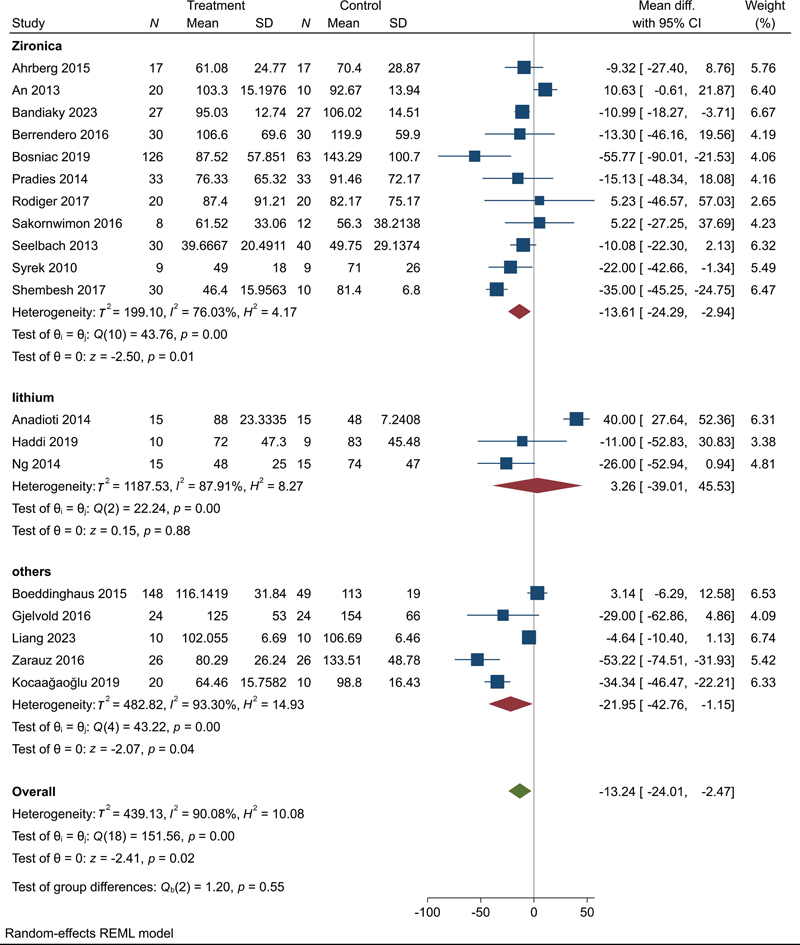
Subgrouping analysis according to the material of restorations.

We found a significant difference between digital and conventional impressions with an overall MD = –13.78 (95% CI: –24.77, –2.79, *p* = 0.01) suggesting that restorations fabricated from digital impressions exhibit better marginal fit compared with those from conventional impressions. Significant heterogeneity was observed among the included studies ( *I* ^2^ = 90.74%) ( Fig. 4 ).

Galbraith chart ( Fig. 5 ) and funnel plot ( Fig. 6 ) were utilized to display the distribution of effect values to evaluate the potential for publication bias.

### Subgroup Analysis

Subgroup analysis was conducted to explore potential sources of heterogeneity and examine the influence of different digital impression systems on marginal fit outcomes.

According to the study design, we subgrouped the studies into RCT, *in vivo* , and *in vitro* . We found that the results of RCT prove that it produces a significant superiority while laboratory studies, *in vivo* and *in vitro* , showed nonsignificant results with MD = –15.02 (95% CI: –27.38, –2.66, *p* = 0.02), MD = –25.07 (95% CI: –59.39, 9.25, *p* = 0.15), and MD = –8.09 (95% CI: –28.31, 12.14, *p* = 0.43), respectively. Also, significant heterogeneity was observed among the three groups with *I* ^2^ = 75%, *I* ^2^ = 57.38%, and *I* ^2^ = 95.83, respectively ( Fig. 7 ).

According to the most frequent techniques used in digital and conventional impressions, we noticed that the Trios' intraoral scanner, CAD, and iTero techniques revealed no difference between it and conventional with MD = –5.83 (95% CI: –12.31, 0.66, *p* = 0.08), MD = –12.25 (95% CI: –5.96, 32.47, *p* = 0.13), and MD = 25.98 (95% CI: –7.45, 59.42, *p* = 0.59), respectively. Significant heterogeneity with *I* ^2^ = 37.11%, *I* ^2^ = 74.02%, and *I* ^2^ = 97.11%, respectively, was observed. Cerec Omnicom scanner versus conventional revealed no superiority too with MD = –29.86 (95% CI: –53.68, –6.03, *p* = 0.01) with major heterogeneity ( *I* ^2^ = 84.01%). Lava C.O.S. (chairside oral scanner) scan versus conventional revealed better results in the Lava C.O.S. group with MD = –25.44 (95% CI: –41.66, –9.22, *p* = 0.01). High heterogeneity was observed ( *I* ^2^ = 59.12%) ( Fig. 8 ).

Lastly, we performed subgrouping according to the most frequently used materials for restorations and we found that digital impressions revealed a significant improvement in the marginal gap for zirconia restorations with MD = –13.61 (95% CI: –24.29, –2.94, *p* = 0.01). Major heterogeneity was observed ( *I* ^2^ = 76.03%) ( Fig. 9 ).

## Discussion

The current systematic review and meta-analysis sought to comprehensively assess the marginal fit of all-ceramic fixed restorations made from digital and conventional impressions.

Our findings suggest that restorations generated from digital impressions exhibit a superior marginal fit compared with those produced via conventional impression procedures.

This aligns with several previous systematic reviews and meta-analyses that have investigated the marginal fit of dental restorations fabricated using digital versus conventional impression techniques. For instance, Chochlidakis et al found that digital impressions are associated with improved marginal adaptation in fixed dental prostheses. 38 39

Tsirogiannis et al conducted a meta-analysis comparing the marginal fit of single crowns fabricated from digital and conventional impressions and reported no significant differences in the marginal gap of single-unit ceramic restorations fabricated after digital or conventional impressions. During the data extraction revision, we encountered limitations related to inconsistent reporting practices. For instance, some studies included the total number of patients, while others focused on the number of teeth. This discrepancy in reporting methods can introduce challenges in data analysis and interpretation, highlighting the importance of standardizing reporting criteria across studies to ensure accurate and meaningful comparisons. 40

Furthermore, our subgroup analysis based on the study design yielded interesting insights. RCTs consistently showed a significant superiority of digital impressions in marginal fit. However, both *in vivo* studies, which are considered as a real-world clinical scenario, and *in vitro* studies did not exhibit a significant difference between the two methods. While RCTs provide strong evidence, clinicians must account for various aspects of clinical practice, the operator's abilities, and patient differences. Future research should focus on specific clinical scenarios and long-term outcomes to help guide evidence-based decision-making.

Digital impressions and CAD/CAM systems offer significant advantages over traditional methods in dentistry. 41 They not only provide exceptional accuracy for precise restorations and improved treatment outcomes but also enhance marginal fit compared with conventional techniques. Intraoral scanners have the potential to replace traditional impressions entirely, especially for fixed denture fabrication, resulting in reduced operating time and increased patient comfort. Patients benefit from a more comfortable experience without the inconvenience of messy impression materials. Furthermore, digital workflows streamline communication between dental professionals and laboratories, leading to greater efficiency and environmental sustainability by eliminating the need for physical materials. 6 11 12 13

However, despite the converging evidence supporting the advantages of digital impressions in enhancing marginal fit, it is crucial to acknowledge the limitations inherent in both the current study and previous systematic reviews. One notable limitation is the heterogeneity observed across included studies, which can introduce variability in outcomes and affect the reliability of meta-analytic results. Heterogeneity may stem from differences in study designs, participant characteristics, types of restorations evaluated, digital impression systems utilized, and measurement techniques employed to assess marginal fit. In a comprehensive evaluation of digital and conventional impression techniques, we performed a subgrouping analysis to compare several intraoral scanners regarding their impact on marginal fit as mentioned in Fig. 6 . We found that the Trios' intraoral scanner did not display a noteworthy distinction in marginal fit when compared with VPS. Similarly, the Cerec Omnicom scanner did not demonstrate a clear advantage over VPS. Notably, the Lava C.O.S. group showcased superior outcomes in contrast to VPS, indicating a potential benefit of using this specific digital impression system for achieving better marginal fit in fixed prosthetic restorations.

Recent studies discussing Trios intraoral scanners found it to be the most accurate single-tooth scanner compared with standard impressions. 42 43

Dauti et al compared Lava C.O.S. digital and conventional impression methods for manufacturing copings, finding no significant difference in marginal parameters between the two groups. Mean marginal gap values for both groups fell within clinically acceptable ranges, indicating that digital impressions can produce copings with similar accuracy to conventional methods. 44 45

In 2018, a systematic review evaluated the precision of intraoral scanning systems such as Cerec Bluecam, Omnicam, iTero, Lava C.O.S, Trios, and others in dental impression accuracy when compared with traditional methods. The review indicated that these systems are dependable for diagnostic and short-span scanning but may exhibit more variation in whole-arch scanning. Various intraoral scanning systems demonstrated differing levels of accuracy, showing promising outcomes yet remaining susceptible to inaccuracies. 46

Zirconia restorations are essential in contemporary dentistry because of their durability and pleasing appearance. 47 48 We found that digital impressions were better for zironic restorations, while there were no significant differences in using digital or conventional impressions for other materials.

Numerous strengths are considered in our meta-analysis. First, we included many recent clinical trials that are related to our topic. Second, we performed a comprehensive analysis and subgrouping for all possible groups that gave valuable insights and new considerations to be done in the future. Third, the articles exhibited diversity in terms of different factors, making it difficult to directly compare the results. We have utilized various methods to address this diversity and thoroughly investigate the potential reasons behind the significant variations.

## Conclusion and Recommendations

This systematic review and meta-analysis provide strong evidence that all-ceramic fixed restorations fabricated using digital impression systems exhibit superior marginal fit compared with those produced through traditional impression techniques. Despite some heterogeneity and methodological limitations in the included studies, our findings align with prior research, demonstrating a consistent trend toward improved marginal adaptation with digital workflows.

Given the critical importance of achieving optimal marginal fit for the long-term success and durability of dental restorations, the following recommendations are proposed:

1. *Clinical adoption* : Clinicians are encouraged to adopt digital impression systems to improve the marginal fit of all-ceramic restorations. These systems offer enhanced accuracy, efficiency, patient comfort, and reduced material waste. However, successful implementation requires adequate training and familiarity with digital workflows.
2. *Standardization in research* : Future studies should adopt standardized protocols for measuring and reporting marginal fit parameters, such as marginal gap and cement thickness, to ensure comparability across studies. Validating digital measurement techniques against gold-standard methods is essential for ensuring accuracy and reliability.
3. *Ongoing innovation and evidence updates* : Continuous monitoring of advancements in digital dentistry, particularly digital impression systems, is essential for clinicians and researchers. Regular updates to systematic reviews and meta-analyses are necessary to incorporate emerging evidence and maintain evidence-based practices. Collaborative efforts among researchers, clinicians, and industry stakeholders are crucial for driving innovation and enhancing dental care quality.

By integrating these findings into clinical practice and research, the potential benefits of digital impression systems can be fully realized, contributing to improved patient outcomes and advancing the field of restorative dentistry.
